# A review of mathematical models of influenza A infections within a host or cell culture: lessons learned and challenges ahead

**DOI:** 10.1186/1471-2458-11-S1-S7

**Published:** 2011-02-25

**Authors:** Catherine AA Beauchemin, Andreas Handel

**Affiliations:** 1 Department of Physics, Ryerson University, Toronto, ON, M5B 2K3, Canada; 2 Department of Epidemiology and Biostatistics, College of Public Health, University of Georgia, Athens, GA 30602, USA

## Abstract

Most mathematical models used to study the dynamics of influenza A have thus far focused on the between-host population level, with the aim to inform public health decisions regarding issues such as drug and social distancing intervention strategies, antiviral stockpiling or vaccine distribution. Here, we investigate mathematical modeling of influenza infection spread at a different scale; namely that occurring within an individual host or a cell culture. We review the models that have been developed in the last decades and discuss their contributions to our understanding of the dynamics of influenza infections. We review kinetic parameters (e.g., viral clearance rate, lifespan of infected cells) and values obtained through fitting mathematical models, and contrast them with values obtained directly from experiments. We explore the symbiotic role of mathematical models and experimental assays in improving our quantitative understanding of influenza infection dynamics. We also discuss the challenges in developing better, more comprehensive models for the course of influenza infections within a host or cell culture. Finally, we explain the contributions of such modeling efforts to important public health issues, and suggest future modeling studies that can help to address additional questions relevant to public health.

## Introduction

The influenza A virus causes annually recurring epidemic outbreaks, most people become infected multiple times over their lifetime [1]. The virus also has the propensity to cause occasional pandemics with potentially high death tolls [2,3]. Influenza infection results in the desquamation of the epithelial cells lining the nasal mucosa, the larynx, and the tracheobronchial tree. In the case of typical, uncomplicated influenza in humans, the infection will involve only the upper respiratory tract and the upper divisions of bronchi [4]. In very severe, and often fatal cases of influenza, the infection will spread to the lower lungs as observed, for example, in some infections with avian influenza strains [5,6]. The site of infection, namely the airway epithelium, consists of a single layer of cells everywhere except in the trachea [7] and is composed of four major cell types: basal (progenitor), ciliated, goblet, and Clara cells [8]. While human-adapted, seasonal strains of influenza tend to preferentially bind and infect nonciliated cells, avian-adapted strains appear to prefer ciliated cells, which could explain these strain’s propensity to infect the lower respiratory tract [6,9-11].

**Figure 1:**
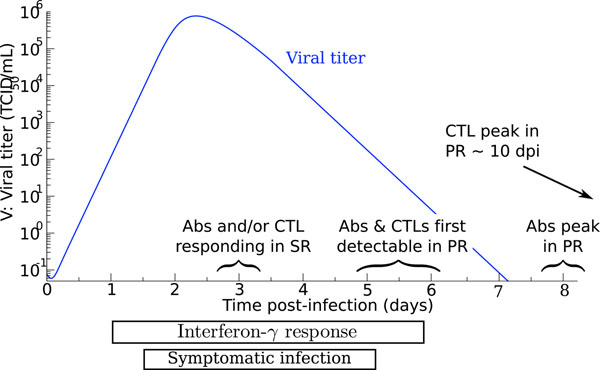
**Course of an influenza infection within a host.** The timings of the adaptive immune response, namely Antibodies (Abs) and cytotoxic T lymphocytes (CTL), for both a primary (PR) and secondary (SR) response to an influenza infection are indicated.

An influenza A infection is typically initiated following the inhalation of respiratory droplets from infected persons. These droplets containing influenza virions (virus particles) first land on the mucus blanket lining the respiratory tract [7,12]. While many virions are destroyed by non-specific clearance such as mucus binding, the remaining virions escape the mucus and attach to receptors on the surface of target epithelial cells. The incubation time for influenza is typically about 48 h, but will typically vary between 24–96 h, possibly owing to the size of the initial inoculum [7]. Cell infection is initiated by adsorption of the virions to the cell surface. The influenza virus hemagglutinin (HA) is responsible for binding the sialic acid receptors on the surface of epithelial cells providing a strong bond, facilitating the virion’s adsorption into the cell. This results in receptor-mediated endocytosis of the virus particles approximately 20 min after infection [7]. Once inside the cell, the virions begin replicating, using the machinery and building materials that would normally be used by the host cell to maintain its functions. Virus budding, which takes place only at the apical surface membrane of infected cells [13], can be detected 5–6 hours post-infection (hpi), and is maximal 7–8 hpi (see Table 1). The period between successful infection of the cell and the productive release of viral progeny is often called the “eclipse phase”. Just as it did upon cell entry, the HA on the surface of the virions will again bind the sialic acid receptors. The virus neuraminidase (NA) is responsible for cleaving the sialic acid receptors on the surface of the cells to allow the newly-produced influenza virions to break free of the cell that has produced it and go on to infect other cells. Successive cycles of cell infection quickly result in an exponential growth of viral titer, which peaks around 2–3 days post-infection (dpi). The infection typically resolves in 3–5 dpi, and virus can typically be isolated between 1–7 dpi [7]. In a primary infection with influenza, pathogen-specific antibodies (Abs) and CD8^+^ cytotoxic T lymphocytes (CTL) are first observed around 5 dpi, peaking around 7 dpi, whereas in a secondary infection Abs and CTLs can respond as early as 3 dpi [14]. Cellular regeneration of the epithelium begins 5–7 dpi but complete resolution can take up to one month [15]. Figure 1 illustrates the kinetic of the course of an influenza infection within a host.

**Table 1:** Kinetic parameters for influenza obtained from both fitting mathematical models to data and by direct estimation from experimental data.

Parameter	Values [References]
Mathematical models to fit experimental data	

Average lifespan of an infected cell	39h [42], 6h and 11.4h [15], 18h and 48h [39], 6h–14h [47], 17h and 40h [31], 1.8h and 33h [45] 28h.
Average infectious lifespan of a virion	111h [42], 4.6h and 8h [15], 8h and 300h [39], 9.5h [47], 1.8h–9.1h [45], 5.7h and 2.6min [31]
Length of the latent (eclipse) phase	6h [15], 0.22h–6h [47], 6h–8.5h [45]
Rate of epithelial cell (re)growth per day	0.72 [42], 0.015 [75], 6.2 × 10^-8^ and 0.34 [31]
Drug efficacy	0.97 and 0.99 [39] (oseltamivir), 0.56–0.92 [47] (amantadine)
Lifespan of interferon	3.5h [74], 60h [75]

Direct experimental measures	

Average lifespan of an infected cell	12–48h [80-86]
Average infectious lifespan of a virion	0.5–3h [87-90]
Length of the latent (eclipse) phase	3–12h [42,80,82-84,87,88,91-93]

Lifespan is defined as the inverse of the rate parameters (the sometimes alternatively used half-life contains an extra factor of log(2)). Note that some of the studies are *in vitro* and some in vivo. Multiple values come from either differences in strains or different models analyzed within a single study. Caveats about the reliability of the estimates obtained from model fitting are discussed in the section titled “Data diversity and quantity and its effect on parameter identifiability”.

Several aspects of influenza infections are still unresolved. For instance, the contributions of strain-specific cell tropism, pre-existing immunity, and host genetic factors in shaping the virulence and transmissibility of a particular influenza strain are not well understood [16,17]. There is much to be learned about how a strain’s genotype shapes complex phenotypes such as virulence and transmissibility. Most of these unresolved aspects will require a quantitative analysis of the key players and of the significance of their respective contribution. As we enter the era of quantitative virology and immunology, with ever more sophisticated experimental tools collecting ever increasing amounts of data, there is more than ever a need for greater synergy between experiments and analysis.

Much work has been done on attempting to capture the dynamics of influenza A using mathematical models; almost all of these models are on the host population level and are concerned with transmission between infected hosts. These models can be used as tools to inform public health decisions with respect to pandemic planning: whom, how and when to quarantine, vaccinate, treat with antivirals, and how much and what to stockpile [18-26].

Here, we focus on a lesser known application of mathematical modeling to the study of influenza kinetics, that aimed at understanding and quantifying the processes involved in determining the severity, duration, and outcome of the progression of the infection within a host or a cell culture. These types of models provide information of a different nature, but, as we will outline below, the information they provide can be equally critical for better treatment and management of the disease. Furthermore, the development of reliable within-host models is critical to improving epidemiological models since the latter relies on the former to more accurately capture the diversity of infection severity, latency, and symptoms.

We first present a survey of the published literature on within-host and in vitro modeling of influenza infections (see also [27] for a recent review of some of those modeling efforts). We then discuss in general terms both the contributions made by those models and the lessons learned, as well as the challenges that remain as we seek to further our understanding of influenza kinetics within a host or cell culture. We close by highlighting the importance of the models to public health and promising directions for additional modeling studies.

## Mathematical models of within-host influenza dynamics and their contributions

### Simple models without an immune response

#### Overview of the models

The most basic models considered to capture the dynamics of influenza infections, both in vivo and in vitro, consist of sets of ordinary differential equations (ODEs), namely

These models describe the dynamics of susceptible target cells, *T*, which become infected at rate *β* by the free virions, *V* . In model (1), the newly infected cells, *I*, immediately begin to produce virus, whereas in model (2), newly infected cells first undergo a latent or eclipse phase, *E*, before they become infectious, *I*, after an average time 1*/k* has elapsed. Infectious cells, *I*, are assumed to produce virions at a constant rate *p*, until they undergo apoptosis after an average time 1*/δ*. Finally, virus produced by infectious cells is eventually lost after an average time 1*/c* due to clearance mechanisms that include loss of infectivity (if the viral titer is measured in units of infectious virus, e.g., pfu, TCID_50_), and binding with antibodies or mucus when analyzing in vivo experiments. Note that these models make the assumption of exponentially distributed latent and infectious periods, which were shown to be incorrect as they cannot reproduce the kinetics of certain experimental influenza infection assays (see Applications to in vitro systems). The use of more appropriate distributions in implementing these delays can alter the model behavior and estimates obtained from data fitting [28-30].

**Figure 2:**
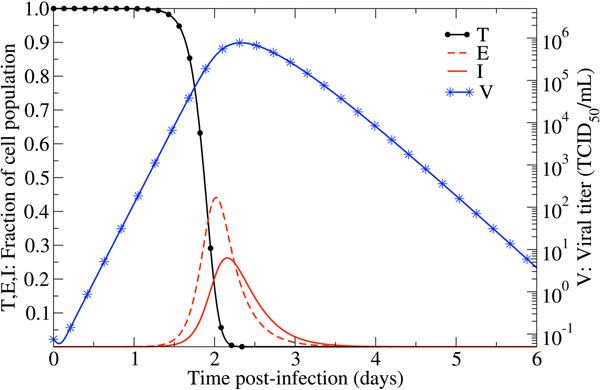
**Typical kinetics exhibited by the target-cell limited model with a latent phase predicting the course of an influenza infection within a host**. We can see that the target cells (*T*) are consumed rapidly, with viral titer (*V* ) peaking shortly thereafter. In this picture, there is approximately a 3*.*6 h delay between the infectious (*I*) cells’ peak and that of viral titer. The parameters, (*β, k, δ, p, c*) = (3*.*2 *×* 10*^-^*^5^ ([V] *·* d)*^-^*^1^*,* 4*.*0 d*^-^*^1^*,* 5*.*2 d*^-^*^1^*,* 4*.*6 *×* 10*^-^*^2^ [V]*/*d*,*5*.*2 d*^-^*^1^), and initial conditions, (*T, E, I, V* )*_t_*_=0_ = (4 *×* 10^8^*,* 0*,* 0*,*7*.*5 *×* 10^2^ [V]), where [V] is TCID_50_/mL of nasal wash are from Table 3 of [15].

The typical kinetics of these models is illustrated in Figure 2 for the model including a latent phase using the averaged parameters presented in Table 3 of [15]. These parameters correspond to the geometric average of a nonlinear fit of model (2) to viral titer from 6 human volunteers infected with influenza A/Hong Kong/123/77 (H1N1). Viral titer grows exponentially, peaks around 2–3 dpi, before decaying exponentially. Target cells are consumed rapidly, with the population of infected cells peaking around the same time as viral titer.

Because this type of model does not explicitly incorporate an immune response (IR), it is said to be target-cell limited, i.e. the virus load reaches its peak and subsequently declines once most cells have been infected and few susceptible cells remain. More accurately, the peak is reached when *βTV* ≈ *δI*. The target-cell limited nature of the models is clearly illustrated in Figure 2 where most target cells have been depleted by 54 hpi, around the time of viral titer peak. This almost complete depletion of target cells needs to be understood in the context of susceptibility: Cells susceptible to the virus and able to produce progeny virions as described by the model do not necessarily directly correspond to all epithelial cells in the respiratory tract. Indeed, it is not well known which cells contribute to the infection dynamics in the way described by the model. This likely varies between different influenza strains and hosts. Some cells might not be susceptible or be productively producing virus for instance due to reduced affinity of the virus for the types of receptors the cells express on their surface [6,9-11], or due to the protection provided by the presence or emergence of an IR not explicitly represented in the model.

#### Applications to in vivo systems

The absence of an explicit IR in target-cell limited models is equivalent to assuming that either the effect of the IR on viral titer levels is negligible, or that its effect is somewhat constant through the course of the infection. In the latter case, the immune system can then implicitly be taken into account through parameters *δ* and *c*, which control the rate of loss of infectious cells (*I*) and virus (*V* ), respectively. And as mentioned above, the target-cell limitation itself can also act as an implicit IR by limiting the number of cells available for infection owing to the protective effect of the IR. Since viral titer in an influenza infection peaks around 48 hpi, whereas the primary adaptive IR is not detectable before approximately 5 dpi [31,32], it might be a reasonable assumption to ignore the IR, though its role on infection clearance is still not fully resolved. We will return to this point later when we discuss models which incorporate an IR.

To our knowledge, the first mathematical model proposed to describe the within-host dynamics of an influenza infection was introduced by Larson et al. in 1976 [33]. The 7-compartment, 5-parameter model was fitted against the viral titer for mice infected with A/Aichi/2/68 (H3N2). The model could successfully reproduce the viral titer curves for virus sampled from the lung, the trachea, or the nasopharynx. While most of the five parameters could not directly be related to specific infection mechanisms (e.g., viral production rate, infected cell lifespan), two of them were of particular interest. Parameter *P*_1_, which corresponds to the initial viral titer (*V* at time *t* = 0) is of interest because the compartment (lung, trachea, or nasopharynx) with the largest initial viral inoculum likely corresponds to the primary area of deposition of the infectious dose administered. Parameter *P*_2_, which corresponds to the exponential viral titer growth rate is of interest because the compartment with the largest viral titer growth rate is that in which the virus reproduces most effectively. The fit of the model to the various viral titer curves indicated that virus replicated most effectively in the trachea, then in the lungs, with the poorest replicative efficiency found in the nasopharynx. Unfortunately, the viral titer sampling was sparse (every 24 h) often providing only one or two viral titer points from which to characterize the initial viral inoculum (*P*_1_) and the exponential viral titer growth rate (*P*_2_). Yet this work shows the early interest in mathematical modeling, and the promise it holds to characterize infection kinetics in a more quantitative way.

Thirty years after the Larson et al. model, Baccam et al. performed a study where they fitted a set of simple differential equation models to experimental viral titer for the course of an influenza infection within a host [15]. They first applied the target-cell limited models (1) and (2), which had previously been applied successfully to HIV [34,35] and HCV [36-38], to fitting viral titer of primary infection of human volunteers with influenza A/Hong Kong/123/77 (H1N1) [15]. Because the variables (target cells, latently infected cells, infectious cells, and viral titer) and parameters (e.g. viral clearance rate, cell lifespan) of the models correspond directly to biological processes, the parameter values obtained from the fits of the models to viral titers provided novel, quantitative information about the kinetics of the infection. While the reported best fit parameter values in this study largely agree with known biology (e.g., Table 1), the values should be used with caution due to the problem of overparametrization, which we will discuss below. It is also important to note that the authors did not perform a sensitivity analysis on the parameters of their models. A year later, Handel et al. also used a simple target-cell depletion model to fit human influenza data in the context of a study of neuraminidase inhibitor resistance emergence [39]. However, the study suffers from the same overparametrization issue as [15]. In addition, since the main focus of that study was not parameter estimation, the authors did not perform as careful an analysis as was done in [15]. For instance, no confidence intervals for parameter values were provided. Since then, additional modeling studies have been performed which incorporated components of the IR with varying levels of details. We discuss these models below.

More recently, Dobrovolny et al. [40,41] have considered a simple extension of model (2) in which two cell populations are represented: a default and a secondary population. The two target cell model can capture the kinetics of uncomplicated infections as well as that of sustained and/or severe infections by incorporating the IR in an implicit way via the secondary cell populations. The default cell population is used to represent the readily accessible cells typically consumed by an influenza infection. The secondary population represents cells that are protected from infection in the case of seasonal infection, but are consumed to varying degrees in severe or chronic infections, perhaps due to differing cell tropism, lack of pre-existing immunity, or an aberrant IR. The two target cell model can also be applied to the study of the effect of cell tropism (different virus strains having different preferences for different cell populations) in cell cultures such as human tracheobronchiolar epithelium (HTBE) cells.

#### Applications to in vitro systems

While models that ignore host factors such as the adaptive IR constitute an approximation of in vivo systems, they more accurately describe in vitro infections. In vitro experiments have long been used to carefully characterize specific aspects of the infection process, which could not be studied easily in vivo. The application of mathematical models to the analysis of in vitro infection systems allows for simple models, which can focus on the kinetics of cell-virus interactions alone, without the need to additionally consider a wide array of host factors, such as the IR.

Several studies of mathematical modeling of in vitro influenza infections, combined with experiments, have been undertaken by the group of Reichl and colleagues [42-45]. They have focused their attention on studying the growth of influenza virus within microcarrier cell cultures, with the goal of characterizing and maximizing viral titer yield in these systems meant to produce virus for use in influenza vaccines. In 2005, Möhler et al. proposed a simple model for the kinetics of infection in a microcarrier of MDCK cells infected with an equine influenza A (H3N8) virus [42]. The model is similar to model (1), but includes the death and regeneration of target (uninfected) cells, the loss of virus due to adsorption onto target cells, and incorporates a fixed delay of 4*.*5 h between cell infection and the start of viral production, instead of an explicit eclipse phase as in model (2). The model in [42] provides a good fit to HA titer data. From their study, the authors conclude that viral yield in these systems can be most effectively maximized by increasing the total number of susceptible cells, upregulating viral production rate, and delaying the apoptosis of infected cells. In a follow-up study, Sidorenko et al. developed a Monte Carlo model for viral titer growth in microcarrier MDCK cultures infected with an equine influenza A (H3N8) virus that incorporates both intra- and inter-cellular infection kinetics. Rather than explicitly representing all aspects of intra-cellular viral replication (a topic the group addressed in a separate study [46]), they characterize a cell’s intracellular infection state as different classes representing cells that contain different numbers of intra-cellular virus equivalents. This allowed the authors to fit not just the viral HA titer, but also the fluorescence distribution for the population of infected cells measured through flow cytometry of cells stained using antibodies against the virus M1 and NP. These and further studies by this group [42-45] offer a unique look at the process of influenza viral infection in microcarrier cell cultures and a rare opportunity to develop and refine intra-cellular models for influenza viral replication by providing high quality experimental data.

The analysis of in vitro data using mathematical models can also reveal infection parameters buried within experimental data. For example, in 2008, Beauchemin et al. used models (1) and (2) to analyze the viral titer over the course of experimental infections of MDCK cells with influenza A/Albany/1/98 (H3N2) in a hollow-fiber reactor under different concentrations of the antiviral drug amantadine [47]. The aim of the work was to characterize the effect of amantadine treatment on the course of the infection. Using different variants of the target-cell limited mathematical models, Beauchemin et al. were able to determine the IC_50_ (0.3–0*.*4 µM) and maximum efficacy (56–74%) of amantadine at blocking the infection of susceptible cells. Research by Beauchemin and colleagues has also focused heavily on the application of mathematical models to the analysis of in vitro infections, with special attention to the properties of in vitro assays. In Holder et al., two mathematical models were constructed to reproduce the course of an influenza infection in two different viral assays: a plaque and a viral yield assay [48]. The aim of the project was to determine if and how the fitness of the oseltamivir-sensitive wild-type (WT) A/Brisbane/59/2007 (H1N1) differs from that of its H275Y (N1 notation) oseltamivir-resistant counterpart. Interestingly, while the plaque assay suggested that the WT strain was fitter (exhibited a more rapid plaque growth), the viral yield assay suggested that instead the H275Y mutant was fitter (exhibited a larger exponential viral growth rate). Using mathematical equivalents of the assays to run a large number of simulated experiments, Holder et al. uncovered that plaque assays, due to the spatial restriction of the overlay on infection spread, were most sensitive to the length of the cell’s eclipse phase, whereas the viral yield assay was equally sensitive to virus infectivity, viral production rate, and the length of the cell’s eclipse phase. This difference in the sensitivity of the assays to different aspects of the viral replication cycle explains why the two assays appear to provide contradictory conclusions about the fitness of the two strains. Thus, mathematical models can help shed light on the limitations or caveats of in vitro assays. In return, in vitro assays can teach us about fallacies in the formulation of our mathematical models. Holder et al. investigated how different in vitro assays can inform model development [30,49]. Notably, they showed that use of either exponential or Dirac-delta distributions for the times spent by cells in the latent or productively-infected state are not consistent with experimental results from single-cycle viral yield experiments, whereas normal and lognormal time distributions are. From these studies, it is clear that a greater synergy between experiments and mathematical models is highly desirable.

### More extensive models which incorporate an immune response

#### The importance of the immune response to influenza infections

As discussed earlier, the kinetics of the viral titer over the course of an influenza infection is well captured with a simple model that does not include an IR. Instead, one can account for the decline in viral load by attributing it solely to the complete depletion of target cells. While complete cell depletion — even if restricted to a localized patch of cells — may appear excessive, at least one histological study of influenza infection of ferrets supports this idea. In [50], Francis and Stuart-Harris examined the lungs of ferrets infected intranasally with a sub-lethal inoculum of influenza virus and noted desquamation of the tracheal area by 2 dpi, which progressed to a complete destruction of the epithelium. Despite this severe insult, the animals survived and their epithelial tissue fully regenerated within a few weeks. While ferrets are generally considered a good animal model for human influenza infections, it is not clear how applicable this result is to influenza infections in humans [51]. Reports of immunocompromised patients shedding influenza virus for prolonged periods suggest that the IR plays an important role in clearing infection, or at least in preventing chronic and potentially fatal outcomes [52-55]. The IR is likely to be especially important in more severe influenza cases where infection also involves the lower respiratory tract [56,57], as observed in infections with avian-origin H5N1 [58,59], and 1918 pandemic influenza strains [60,61]. In fact, it is quite likely that some of the virulence of influenza strains such as H5N1 or the 1918 H1N1 is due to an over- or mis-responding IR causing immunopathology, though the details are yet to be resolved [62-66]. Further evidence of the crucial role of the IR comes from animal studies in which components of the IR are depleted. Those studies taken together suggest that both innate and adaptive IR are important [32,67,68]. Thus, to gain a comprehensive understanding of the progression of an influenza infection within a host, i.e. to understand specifically how host factors shape the course and outcome of an influenza infection, models incorporating an IR are essential. Several recent modeling studies have taken the IR into account, we will discuss those in the next section.

#### Influenza models incorporating an immune response

To our knowledge, the first influenza modeling study that included components of the IR was a very detailed ODE model developed by Bocharov et al. in 1994 [69]. The model tracked macrophages, CD4^+^ T-cells and CD8^+^ Cytotoxic T Lymphocytes (CTL), B-cells, antibodies and interferon (IFN) in a very detailed manner. The authors used the model to analyze how different components of the IR affect infection kinetics. In particular, they found that a 50-fold increase in specific antibodies and CTLs could prevent an infection from occurring. Another model that is similar in detail to the Bocharov et al. model has recently been developed and studied by Hanciglou et al. [70]. The authors analyzed the effect of initial viral load on the infection kinetics and found that for small initial viral load the disease progresses through an asymptomatic course, for intermediate value it takes a typical course with constant duration and severity of infection but variable onset, and for large initial viral load the disease becomes severe. Two other models, by Chang et al. [71] and Tridane at al. [72], are simpler models based on a variant of the target-cell limited model (2), with an additional component to describe the dynamics and effect of CTLs. The Chang et al. study also included IFN. Chang et al.’s model suggested that the time and level of virus peak is influenced by the innate (IFN) response, while the duration of the infection and clearance phase is determined by the CTL response. Tridane et al. focused in their study on investigating the impact of different model choices for the CTL response on the infection dynamics and found that slight changes in how the CTL dynamics is implemented can influence the resulting dynamics.

Some of the results obtained from these models could have important implications for treatment or vaccine strategies. However, large uncertainty with regards to parameter values and overall model structure make it difficult to evaluate the validity of the models and their predictions. While all these models [69-72] were constructed and parametrized based on the known biology of influenza infections, until such models are brought into direct contact with data for model validation or falsification, the results should mainly be considered as providing conceptual insights.

Several other modeling studies have been performed that were based on a direct connection between models and data. In the study by Baccam et al. [15] already mentioned above, the authors employed one model that included an IR component, namely IFN. Fitting such a model to data, they found that while the fits obtained from this model were not statistically significantly better, they could reproduce a double peak in virus load, something that was observed in a few of the patients they studied. In another study dealing with the issue of drug resistance emergence during infection, Handel et al. [39] fitted both a model with and without an IR. The latter included a very simplistic version of an antibody response. The authors found that the available data did not permit discrimination between the two models.

More detailed and comprehensive models that compared various IR models to data have since been published. Lee et al. [73] developed a model that included all the major effectors of the adaptive IR (CD4^+^ and CD8^+^ T-cells, B-cells/antibodies) as well as explicit descriptions of the IR activation process mediated by dendritic cells. They were able to compare their model to (sparse) data and thereby partially validate it. A follow-up study by the same group made use of extensive experimental data specifically collected for the purpose of fitting the model [31]. In this study, Miao et al. used several ingenious ideas for fitting their models to the data. They divided the course of the infection into an early and late phase, corresponding to infection kinetics in the absence and presence of an adaptive IR, respectively. They also did not attempt to construct ODEs to capture the dynamics of IgA and IgG antibodies, and CTLs. Instead, they fitted smooth curves to the data sets, which were then used as given quantities in their ODE model. The separation of infection dynamics into an early and late phase and the large amount of data the authors had available allowed for a more detailed analysis of the importance of different aspects of the IR (innate, adaptive) during the course of the infection, and the authors were able to estimate important kinetic parameters and how they varied during the infection (see Table 1).

Another recent study by Saenz et al. used a unique dataset of virus load and infected cell levels in ponies to study a model that included an IFN response [74]. The study suggested that inclusion of an IFN response was sufficient to describe the dynamics, while a model without it did not fit the data well. It is still possible, and quite likely, that other model alternatives with adaptive IR components might fit the data equally well, therefore the relative importance of IFN is not yet fully established.

Yet another modeling study using data from two different experimental studies in mice with intact and compromised IRs was conducted by Handel et al. [75]. The authors compared different models with and without both innate and adaptive IR components. They found that both the innate and adaptive IRs are required to provide adequate explanation of the data. For one of the datasets investigated, the authors found that they could not describe the IFN data using a simple equation such as those used in other studies [15,74]. Instead, Handel et al. used the IFN level as given and fitted the remaining data to the dynamical model using an approach similar to that used in [31]. The study also showed that a discrimination between different types of adaptive IRs (i.e. T-cell versus B-cell/antibody) was not possible based on the available data [75].

The studies described thus far are based on differential equations. Another class of models, spatial agent-based models, were also proposed [76,77]. In 2005, Beauchemin et al. introduced an agent-based model for the spread of influenza within a host. In 2006, Beauchemin used this model to explore how spatially localized effects can come to shape and dominate the course and outcome of the infection. While the model is more akin to a toy model, its analysis did yield some insight into the effect of spatial versus well-mixed implementation of cell regeneration and CTL expansion on chronic infection outcomes.

## Lessons learned and challenges ahead

### Conceptual insights and parameter estimates obtained from the models

At the most fundamental level, models can be used to explore a complicated dynamical system, and to gain basic insights into the relative importance of host and viral factors. Such models can either be simple and try to capture only the most fundamental interactions making up the kinetics of the infection, or they can be detailed and try to integrate most of the known biological processes. Unfortunately, the quantity and diversity of available data is usually limited and the results from many modeling efforts therefore remain predominantly conceptual and qualitative. These models are still excellent tools that can help shape our understanding of infection kinetics. The power of modeling has been well documented for HIV [78,79]. For influenza, some useful insights have thus far been obtained as well.

The spatial influenza models described at the end of the previous section [76,77], provided important qualitative insights into the importance of spatial effects in the infection dynamics. Another insight was provided in the study by Handel et al. [39], where it was shown that different assumptions for within-host dynamics (a model with and without an IR) lead to vastly different predictions for the likelihood of drug resistance emergence during treatment. This suggests that it will be crucial to better understand the role of target-cell depletion versus IR, an issue that has since been addressed in several more recent modeling studies [74,75]. Another important conceptual contribution was made by the study of Smith et al. [27], which showed that a two-phase approximate solution can be used to characterize virus dynamics, and this concept was applied to fit data in the study by Miao et al. [31].

Once sufficient understanding of a system has been obtained and data are available, one can formulate mathematical models that encapsulate specific mechanistic hypotheses. By comparing the models with data, one can discriminate between those hypotheses. For instance, in the work by Saenz et al. [74], the hypothesis could be phrased as “a model purely based on target-cell depletion does not explain the data, while a model that includes an IFN response can”. By fitting the model to data, the authors were able to test (and affirm) this hypothesis. The crucial part for such studies is the availability of sufficient data to allow *falsification* of models. Just because a model fits the data does not mean that it is correct. This is especially true for more complicated models, such as the ones including detailed IRs. However, if a model does not fit the data, one has learned something important, i.e. that the mechanisms as implemented in the model do not adequately represent what is going on in the real system. Often, data can permit the elimination of certain models and hypotheses, while a large number of usually more detailed models cannot be ruled out. An example of this is the study by Handel et al. [75] mentioned previously. While the authors were able to rule out a model that did not include an innate or adaptive IR, they were not able to discriminate between different alternative implementations of such IRs. It is therefore important to realize that “negative results”, i.e. the lack of agreement between model and data, is often the most important insight.

Once a model has been found that can be trusted to reasonably approximate the biology (i.e. the model is well-supported by a fair amount of data), one can fit the model to experimental data to obtain estimates for the kinetic parameters of a system. This is especially helpful if such parameters cannot easily be obtained through direct experimental measurements. The parameters one can estimate depend on the specific model and the data available. Almost all models published so far include parameters for the average lifespan of an infected cell and the half-life of virions. Other parameters that have been estimated from some models include the average length of the eclipse (latent) phase, the growth or replacement rate of new susceptible cells and the efficacy of drug treatment. In Table 1, we list estimates for some of these parameters, obtained from fitting data to the mathematical models as described above. For comparison, we also show estimates based directly on experimental studies. However, one should be aware of the key caveats regarding the reliability of parameter estimates obtained from fitting models to experimental data. These are discussed in the next section.

### Data diversity and quantity and its effect on parameter identifiability

Quantitative knowledge of infection parameters could provide much needed answers to many important questions. For example, say one could determine quantitatively from experiments the rate at which virions of a given influenza strain are produced in a given cell line. Using this information one could compare strains with respect to their replication efficiency, could map how specific mutations within a given strain affect specifically viral production, or what concentration of an antiviral is required to block viral production by a specific amount. What, then, are the roadblocks in making this goal a reality? One is insufficient data, both in terms of diversity, quality, and quantity. While more complete and complex mathematical models can be developed readily, use of these models to predict infection kinetics or to estimate unknown parameters is questionable if critical aspects of the model or key parameters are unknown or too poorly supported by experimental data. Thus, if one is to use mathematical models to extract parameter values, one can only add as many components as can be determined from data, and this, ultimately, is what limits the complexity of the models [94].

Consider, for example, the target cell limited models (1) and (2). These models are well-suited to the analysis of viral titer curves from in vitro or in vivo uncomplicated infections because, like the models themselves, these curves typically follow a simple shape: a period of exponential viral growth (*λ_g_*), followed by a peak in the viral titer (*V_p_*) at some time *t_p_* post-infection, followed by a period of exponential viral decay (*λ_d_*). By linearizing model (2) about (*T, E, I, V* ) = (*T*_0_*,* 0*,* 0*,*0), Smith et al. [95] derived an expression for *λ_g_* and for *λ_d_*. More recently, Holder et al. [49] simplified the expression for *λ_g_* further by assuming that  such that, for model (2)

These expressions illustrate how the determination of *k*, *δ*, and *c* based solely on viral titer is not be possible. As remarked by Smith et al. [95], this degeneracy is likely the reason why parameter fits obtained in Baccam et al. [15] for different patients often resulted in these three (or two of these three) parameters being set to the same value by the parameter fitting routine.

Ultimately, since viral titer courses can be well described using just four parameters [30], one can at best hope to extract four parameters from viral titer alone. The issue of parameter identifiability for models (1) and (2) has been investigated by Miao et al. [96] who determined for each of the two models what type of data (e.g. viral titer alone, or viral titer and fraction of infected cells) and how much of that data (e.g., 2 points, 8 points) would be required to identify each of the model's parameters. These studies [30,96] outline the need for more data. Not only is more data needed, quality and diversity are also crucial. For example, having 100 viral titer measurements all sampled after viral titer peak simply cannot make up for the absence of points prior and near the viral titer peak without which one cannot infer the shape of the viral titer curve. Equivalently, measuring only virus titer will not be enough to give a full picture, other quantities, such as different components of the IR, will also need to be carefully measured.

### Reconciling the disconnect between experimental measures and model variables

To allow easy comparison with experimental data, mathematical models are typically described in terms of variables, which correspond to, or can easily be related back to, experimentally measurable quantities. Unfortunately, these quantities (e.g., plaque forming units, fluorescence level) are at best relative measures of quantities of interest (e.g., infectious viral titer levels, proportion of cells infected) and are often related to quantities of interest in nontrivial ways. For example, the number of plaque forming units (pfu) in a viral sample is thought to correspond to the number of virions that are infectious to the cells in the culture used to measure the viral pfu. If the virus solution is collected from a ferret but the viral pfu is measured in MDCK cells, one has not determined the number of virions infectious to ferrets, but rather those infectious to MDCK cells. When using a count of the total number of virions rather than the number of infectious virions (using RT-PCR, for example), models typically assume that the fraction of infectious to non-infectious virions over time remains constant. However, since influenza virions loose infectivity faster than they lose RNA integrity [97], it is unlikely that this assumption is correct. Experimental results for HIV suggest that virion infectivity is not constant over time [98] and mathematical models have incorporated this using a time-varying infectivity for virions [99]. Thus, when constructing models with the aim to extract parameters, modelers must be aware of the nature of the measured quantities and have a keen understanding of how they relate to the variables of their model.

The units used to measure the experimental quantities will often “contaminate” the model’s parameters, greatly limiting the usefulness of their values. One way to emphasize the effect of relative measures on a model’s parameters is to perform a rescaling of each variable to see how each parameter will be affected. For example, using model (2), let us consider the rescaling of the cell population *C_m_* = Γ*C* where *C* is any of *T*, *E*, or *I*, and *V_m_* = *γV* , with *C* and *V* the true number of cells and infectious virus particles, respectively, and *C_m_* and *V_m_* their experimentally measured equivalent. Thus, we get

where only the virus infectivity, *β*, and the viral production rate, *p*, are affected by the rescaling. Hence, unless both *γ* and Γ are known, the viral production rate cannot be expressed in terms of useful units, namely number of infectious virions produced per infected cell per unit time. Beauchemin et al. [47] remarked that, in their assays, the measured viral titer corresponded to no more than 10% of the true number of infectious virus particles.

While knowing the relative value of *p* is sufficient in many applications (e.g., strain A produces three times more virus than strain B), the modeling of drug resistance demands that the absolute value of this parameter be known. Indeed, the rate of emergence of mutations is dependent on the rate of production of virions and unless experimental units of infectious virus (e.g. pfu/mL) can reliably be converted to an actual number of infectious virions, model predictions for the emergence of resistance are uncertain and their robustness must be tested using various viral production rates.

It is also important to understand that parameters extracted by applying a mathematical analysis to experimental data can sometime also be extracted from experiments. However, the parameters extracted experimentally may not be equivalent to those extracted through mathematical modeling of experimental data. For example, several in vitro assays are traditionally used to estimate the IC_50_ of a drug against a particular virus strain. In such assays, the IC_50_ represents the drug concentration required to half the viral titer or fraction of dead cells observed experimentally compared to that seen for an untreated infection at a given time post-infection. Since in these experimental assays the IC_50_ is defined as the concentration required to half a certain experimental observable, the IC_50_ estimated in this manner varies between different techniques and assays, and cannot readily be compared. In contrast, mathematical models define the IC50 as the concentration of drug required to half a specific viral replication parameter (e.g. virus production rate by an infected cell) [47]. In that case, the effect of a drug on a virus replication parameter can be measured in several different assays by fitting the mathematical model to assay data (e.g., viral titer versus time for different drug concentrations). The IC_50_ estimates thus obtained are more robust and should be readily comparable for a given cell-virus strain pair, irrespective of the details of the experimental procedure followed. As such, mathematical models may present a preferable approach to extracting the IC_50_ for a given drug-strain pair.

## Discussion

### Public health contributions of mathematical models

The usefulness of mathematical modeling for public health has long been recognized at the between-host population level. Starting as early as the work of Bernoulli [100], and being steadily used since the beginning of the 19th century, all the way to the recent heavy use of detailed models for influenza and other infections, mathematical and computational models have been and continue to play an important role in shaping our understanding of the evolutionary and transmission dynamics of infectious diseases, and are important tools for designing appropriate intervention strategies [18-26,101]. While the mathematical modeling of infection dynamics within a host does not have as long a history as that of between-host modeling, it has nevertheless already contributed important insights. Most notably, several studies combining data with mathematical models for HIV have had a direct impact on drug treatment strategies [34,79,102]. The use of mathematical and computer models to study influenza infection within a host or in vitro is much younger than the modeling of influenza at the population level or the within-host modeling of other viral infections such as HIV. Most of the mathematical modeling of influenza infections has only been pursued in earnest during approximately the last five years. While this means that most results obtained today are tentative and further studies are certainly needed, the models have already provided some important insights. As described above, the models helped to better quantify viral fitness [48], shed light on the importance of specific IR components on the infection dynamics [31,74,75], gave conceptual insights into the role of putative vaccines and virus inoculum dose on virus dynamics and severity [69,70], and allowed for the estimation of drug efficacy [39,47]. While most of these findings clearly need to be confirmed and further strengthened by additional studies, both experimental and theoretical, the direct implications for how we understand the infection and implement better treatment strategies such as vaccinations and drugs, is evident.

### Future directions

It is obvious that a general direction for the future is to perform more and more detailed modeling studies, preferably in close contact with appropriate data (i.e. data of high quality, quantity, and diversity). The recent combined experimental and modeling work by Miao et al. [31] is a very promising step in that direction. Modeling studies can contribute to our understanding of influenza infection dynamics in many different ways, here we just name a few directions that we believe to be especially interesting and important.

While understanding the infection dynamics per se is a useful and necessary first step, in the end we are interested in outcomes that are important from a medical or public health perspective. For instance, can we develop within-host models that can produce and predict quantities such as “virulence” or “transmissibility” as readouts? Several tentative steps in this direction have recently been made [39,74], but confirmation with experiments is currently lacking.

To further improve treatment and intervention strategies, we need to better understand their impact and consequences. With the increasing level of antiviral resistance in circulating influenza strains, much activity is currently ongoing to investigate the usefulness of drug combination therapy for the treatment of influenza, a strategy similar to that already employed with HIV [103-110]. We believe that experimental work in this area will benefit from the additional input, which dynamical models like those outlined here can provide [73].

Another area of interest is to better understand vaccines and vaccine efficacy [111]. Most experimental animal studies currently measure protection by vaccines by recording mortality of the animals. However, it might be useful to have a more nuanced understanding of the effects of vaccine on the infection dynamics, something for which mathematical models are again useful tools [112].

One active area of research in biology in general is the development of multi-scale models [113-117]. The interest in multi-scale models can be seen as driven by an overarching long-term goal: To develop the ultimate predictive tool that would allow one to take a genome sequence of a new influenza strain (or other pathogen), and based on the sequence, predict crucial phenotypes such as virulence, transmissibility at the population level, and susceptibility to drugs – without the need of potentially difficult and lengthy experiments [118,119]. We are obviously far from such a comprehensive framework, but mathematical and computational modeling will be essential to reaching that goal. To make progress in that direction, we need to develop models that allow mappings from genotype to complex phenotypes, which naturally calls for a multi-scale, “systems” approach. As an example, the virus production rate is clearly determined by dynamical processes inside an infected cell. The models described above predominantly keep the rate of viral production by infected cells fixed. The simplest models assume that production starts right away once a cell gets infected (Eqs. (1)), whereas slightly more detailed models allow for an initial latent phase (Eqs. (2)). Recently, Sidorenko et al. [46] developed an intra-cellular model for influenza replication and used it to study virus production rate. Their model suggests that virion production is not constant but instead increases with time. While the lack of good intracellular data means the caveats about quantitative interpretation of the results outlined above apply, it will clearly be interesting to start combining such intracellular models with the cell-population based models described herein. Similarly, it will be important to connect the within-host dynamics with the dynamics at the host population level [113,120]. A few recent studies have started to link those two scales for influenza to study drug resistance [39] and to map within-host virus load to transmission potential [121].

While the development of new and more detailed models and data will be important, it is equally important to improve the rigor with which models are analyzed. For instance, extensive sensitivity analysis, as has been used in infectious disease modeling [122-124], could be useful. This is especially true for models containing many parameters that are not fitted but instead estimated from the literature. More sophisticated, multi-level fitting schemes and Bayesian/MCMC frameworks that go beyond the current simple approaches might prove equally helpful [125-127].

While our focus in this review has been on *dynamical* mathematical and computational models based on differential equations or similar dynamical formulations, another class of models, namely static or statistical models, have also recently contributed insights into influenza infection dynamics. In a very exhaustive study of human experimental influenza infections, Carrat et al. [128] were able to use a combination of data and simple models to estimate the duration of infectiousness and other quantities. Liao et al. [129] reanalyzed the same dataset and used it to estimate the relative transmissibility of different influenza strains, as well as other epidemiologically relevant quantities. Lau et al. [130] used similar approaches to analyze data for naturally acquired infections. A combination of such static/statistical models with the dynamical models described herein will likely to lead to further progress.

### Summary

In our opinion, mathematical and computational models are powerful tools to study the infection dynamics of infections. The last few years have seen increased interest in such modeling studies, and we are likely going to see further increases in such studies in the future. We believe such studies can do for influenza what similar studies have already done for infections such as HIV or HCV. To achieve this, it will be crucial that the models be connected to data as tightly as possible, and that the model type and complexity is appropriate for the question one wants to address. As long as these simple rules are followed, we have no doubt that modeling will continue to provide important insights into the infection dynamics and will eventually help us address several of the questions mentioned in the previous section, as well as many others. In addition, much of the progress will not only benefit our understanding of influenza, but will also help to study other acute infections on the within-host level, an area that is still much less developed compared to similar studies at the between-host level.

## List of abbreviations

IR: Immune response; HA: Hemagglutinin; NA: Neuraminidase; dpi: Days post-infection; hpi: Hours post-infection; CTL: Cytotoxic T lymphocyte; Abs: Antibodies; ODE: Ordinary differential equation; TCID_50_: 50% Tissue culture infectious dose; IC_50_: 50% Inhibitory Concentration; pfu: Plaque forming units; MDCK: Madin Darby canine kidney; IFN: Interferon

## Competing interests

AH declares that he has no competing interests. In the preparation of [40,41], CAAB received financial support from F. Hoffmann-La Roche, Ltd.

## Authors' contributions

CAAB and AH were both responsible for drafting the manuscript and contributed equally. All authors read and approved the final manuscript.
